# Biomechanical Stability of Dental Implants in Augmented Maxillary Sites: Results of a Randomized Clinical Study with Four Different Biomaterials and PRF and a Biological View on Guided Bone Regeneration

**DOI:** 10.1155/2015/850340

**Published:** 2015-04-12

**Authors:** Troedhan Angelo, Wainwright Marcel, Kurrek Andreas, Schlichting Izabela

**Affiliations:** ^1^Institute for Maxillofacial Surgery and Dentistry, General Hospital of Vienna “Hietzing”, Wolkersbergenstraße 1, Pavillon 3a, 1130 Vienna, Austria; ^2^Implantology Clinic Kaiserswerth, Kaiserswerther Markt 25, 40489 Dusseldorf, Germany; ^3^Implantology Clinic Oberkassel, Dominikanerstrasse 10, 40545 Dusseldorf, Germany; ^4^Center for Facial Esthetics Vienna, Brauhausgasse 12, 1050 Vienna, Austria

## Abstract

*Introduction*. Bone regenerates mainly by periosteal and endosteal humoral and cellular activity, which is given only little concern in surgical techniques and choice of bone grafts for guided bone regeneration. This study investigates on a clinical level the biomechanical stability of augmented sites in maxillary bone when a new class of moldable, self-hardening calcium-phosphate biomaterials (SHB) is used with and without the addition of Platelet Rich Fibrin (aPRF) in the Piezotome-enhanced subperiosteal tunnel-technique (PeSPTT). *Material and Methods*. 82 patients with horizontal atrophy of anterior maxillary crest were treated with PeSPTT and randomly assigned biphasic (60% HA/40% bTCP) or monophasic (100% bTCP) SHB without or with addition of aPRF. 109 implants were inserted into the augmented sites after 8.3 months and the insertion-torque-value (ITV) measured as clinical expression of the (bio)mechanical stability of the augmented bone and compared to ITVs of a prior study in sinus lifting. *Results*. Significant better results of (bio)mechanical stability almost by two-fold, expressed by higher ITVs compared to native bone, were achieved with the used biomaterials and more constant results with the addition of aPRF. *Conclusion*. The use of SHB alone or combined with aPRF seems to be favourable to achieve a superior (bio)mechanical stable restored alveolar bone.

## 1. Introduction

Physiology of bone and bone-healing after mechanical trauma (natural and iatrogenic macroscopic and/or microscopic fractures) is well known in general medicine for long [1] and biologically defined by the core function of peri- and endosteum [2, 3] based on its histologic composition [4].

**Figure 1:**
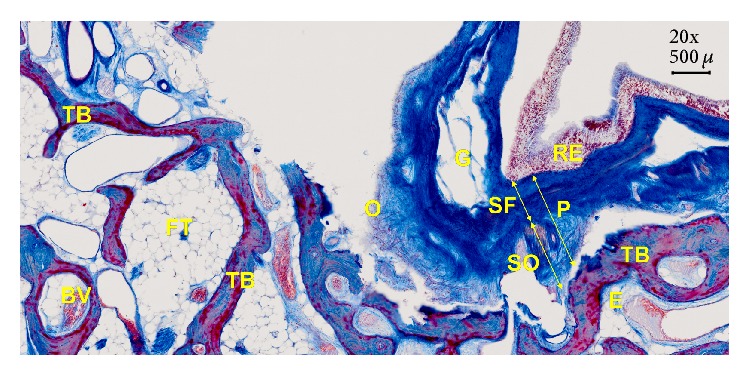
Section through a human maxillary sinus specimen after detachment of the Schneiderian membrane with the tHUCSL-INTRALIFT-method in a fresh human cadaver head. RE: respiratory epithelium pointing inside the maxillary sinus, P: periosteum (total), SF: subsection of the periosteum (P): fibrous layer (“stratum fibrosum”), SO: subsection of the periosteum (P): osteogenic layer (“stratum osteogenicum”), O: osteoblasts (red dots), TB: trabecular bone (reddish), E: endosteum (blue layer covering all TB), G: gland, BV: blood vessel, and FT: fat tissue. Specimen at 20x magnification was prepared by immersion fixation in 5%-neutral-formaldehyde-solution, dehydrated with alcohol, embedded in Paraplast, and cut with a microtome to slices of 5-6 microns. Azan-staining was performed in order to visualize the osteoblasts within the periosteal layer (reddish color), the collagenous fibres of the periosteum and connective tissues of the sinus-membrane, and the connecting Sharpey fibres (dark blue color).

A “de novo” bone formation under the elevated Schneiderian membrane in sinus-lift-procedures [5] even in absence of auto-/hetero-/xenogeneic or synthetic biomaterials [6] was proven experimentally and clinically since the Schneiderian membrane is composed of mainly periosteum, lining the bony walls of the maxillary sinus [7] (Figure 1).

Obviously the known fact of vital peri- and endosteum to be the core carrier of bone healing and regeneration was long neglected in oral surgery and implantology [8], leading to numerous studies focusing on hypothetical osteoinductivity and/or osteoconductivity of auto-, heterologous, and xenogeneic bone grafts versus synthetic biomaterials [9–15]. Only recently it was investigated if the applied surgical technique, for example, sinus lifting, preserved an intact and vital elevated periosteum or merely led to a dissection of the periosteum from the respiratory epithelium [16], which leads to failure in guided-bone-regeneration (GBR) procedures. Contrary, a proven clean periosteal detachment [7] provides higher and constant success-rates in sinus lifting [17] even in the less remodeling-active posterior maxilla [18].

Both from biological and physiological standpoint, sinus lift procedures, regardless if performed with lateral or transcrestal approach, technically have to be considered as “subperiosteal tunnel” or “pocket” techniques, creating a bone-based and enclosed subperiosteal scaffold for guided bone regeneration (GBR) without the need to raise a full thickness mucoperiosteal flap especially in transcrestal procedures [17].

The subperiosteal tunnel technique (SPTT) for reconstruction of atrophic alveolar crests in the maxilla and mandible was first described in 1982 [19] and later evaluated with increasing patient numbers using nonporous hydroxyapatite (HA) ceramics [20] but suffered from displacement of the loose granules in the healing period [21]. Improvements of the technique for more predictable results were presented 1991, using nonporous HA, locked-up in a resorbable Vicryl-tube [22] or microporous HA with mechanical stabilization of the subperiosteal tunnel by a surgical splint [23]. These techniques enhanced the results achieved with SPTT regarding vertical height gain of the atrophic mandible by better immobilization of the synthetic bone graft and allowed a better stability of removable overdentures. Different biomaterials and addition of recombinant human platelet-derived growth factor BB (rhPDGF-BB) were investigated regarding bone regeneration with SPTT [24], matching the results achieved with the same biomaterials and rhPDGF-BB in sinus lift procedures [25] as well as injectable hyaluronic acid-based hydrogels with nanohydroxyapatite [26]. Nevertheless, the known absolute demand for a proper immobilization of a metallic implant into bone beyond a biological threshold to achieve full osseointegration of orthopedic [27, 28] and dental implants [29] was long neglected for guided-bone-regeneration-surgeries (GBR) such as SPTT and has to be applied also to particulate bone grafts used for GBR in oral surgery: they follow exactly the same pattern of osseointegration [30, 31] but might only later undergo resorption and/or replacement by native bone in the natural cycle of bone-remodeling [32].

**Figure 2:**
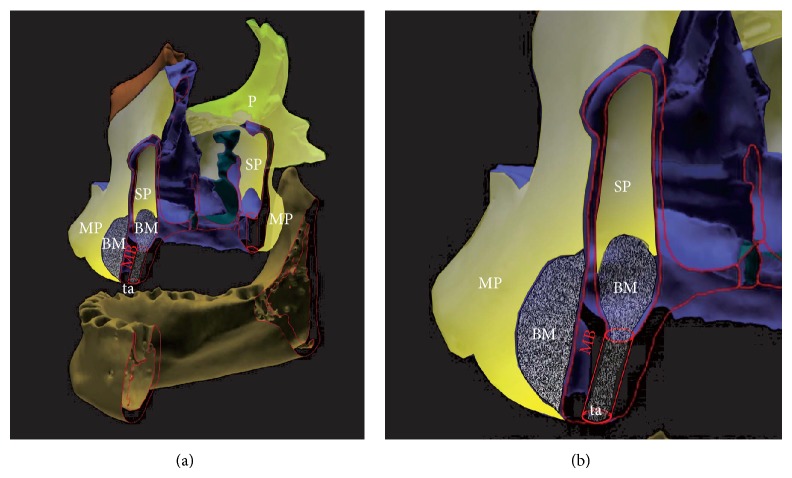
Graphical depiction of anatomical correlation and biological similarity of sinus lift versus buccal subperiosteal tunnel technique-augmentation-sites (overview (a), detail (b); periosteal cover of all bone surfaces depicted in yellow) MB: maxillary bone outlines (red) MP: mucoperiosteum of the oral cavity, SP: sinus periosteum of the maxillary sinus, P: periosteal cover of facial bones, BM: biomaterial applied subperiosteally in sinus lift and subperiosteal tunnel site, and ta: transcrestal approach of the tHUCSL-INTRALIFT-procedure.

When compared, both the minimal invasive transcrestal sinus lift and the subperiosteal tunnel technique provide a subperiosteal scaffold with the bony base of maxillary bone, which is filled then with devital (free) autologous, xenogeneic, or synthetic bone grafts (Figure 2) to create a sufficient future bone-volume and biomechanical stable crestal bone for dental implant insertion. The biomechanical properties of the restored alveolar crest (or subantral augmentation) and satisfactory long-term implant prognosis are expressed best by higher insertion-torque-values (ITVs) determined at dental implant insertion into the augmented alveolar crest (or subantral augmentation) compared to natural maxillary bone [33]. Since a successful restoration of the alveolar crest in the timely healing process after GBR is mandatorily preceded by sufficient angiogenesis and vascularization of the scaffold especially in critical size defects of more than 2.7 mm [34] the highly mechanosensitive nature of this process has to be taken into account strictly [35]: macro- and micromotions of the detached mucoperiosteum by muscular activities in and around the entire oral cavity, around the jawbones and inside the maxillary sinus by breathing activity cannot be avoided at all but might be alleviated by an enhancement of the preceding angiogenesis in the healing cycle. This suggested enhancement was attributed to Platelet Rich Plasma (PRP) [36] or, more recently, to Platelet Rich Fibrin (PRF) [37, 38] also for its properties of enhanced osteogenic differentiation of mesenchymal stem cells originating from the peri- and endosteum [39, 40].

The clinician in his daily routine work therefore has to rely on GBR-techniques, biomaterials, and transplant procedures that provide reliable and reproducible results with possible long-term dental implant success expressed by easily obtained and proven reliable clinical parameters such as the insertion-torque-value (ITV) at implant insertion [41–43].

Aim of the study was to investigate the resulting biomechanical stability of the restored alveolar crest after completed bone regeneration when two different moldable and in situ hardening calcium phosphate bone graft substitutes [30] are used with the subperiosteal tunnel technique in the anterior maxilla to determine if this new class of biomaterials, by their inherent physical property of demanded immobilization [35] of the augmentation scaffold, could achieve consistent results expressed by comparable or higher ITVs than natural maxillary bone and the influence of Platelet Rich Fibrin (PRF) [31] when added to the bone-block-like biomaterial. The results then were compared with the results of a similar study [33] investigating the mechanical quality of restored subantral bone using four different long-used and well documented biomaterials in transcrestal sinus lifting as to possibly demonstrate the suggested biological and clinical similarity between these two different surgical techniques.

## 2. Material and Methods

82 consecutive regular patients eligible for guided bone regeneration in the anterior maxilla from incisor to canine region with sufficient alveolar crest height of a minimum of 14 mm and less than 3 mm alveolar crest width were treated with the Piezotome enhanced subperiosteal tunnel technique (PeSPTT). The patients were aged between 29 yrs and 71 yrs.

82 subperiosteal tunnel sites from the maxillary first incisor to the canine region were augmented in these patients and 109 implants were inserted after the healing period. All patients were antimicrobial shielded with either Amoxicillin/Clavulan Acid 1 g 2×/day or Clindamycin 300 mg 3×/day for 5 days, starting one day before surgery.

26 patients presenting an alveolar crest height of a minimum of 14 mm and minimum width of 5 mm served as control group of natural maxillary bone in the front-to-canine region receiving the same implants as the study groups (single stage Q1-Implant, 3.5/14 mm, TRINON Karlsruhe GmbH/GER) resulting in 30 anterior maxillary implant sites (*n* = 30).

Since subperiosteal tunnel technique is a scientifically well-established and documented surgical procedure and the chosen biomaterials are certified in the European Union, no approval from an ethical committee was necessary according to EMEA guidelines for this clinical study. Every patient signed the consent to receive the CE-certified biomaterial-variant randomly as well as possibly advanced Platelet Rich Fibrin (aPRF), obtained from autologous blood.

**Figure 3:**
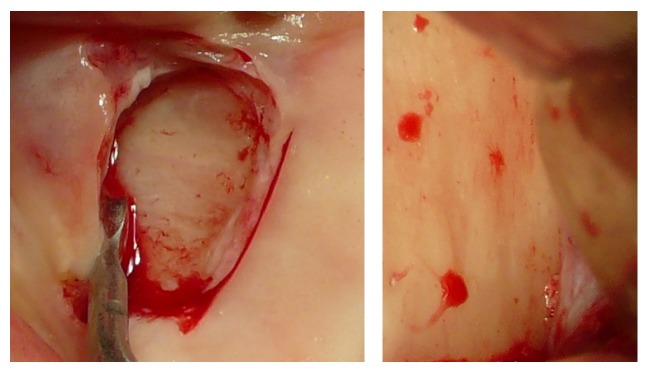
Macroscopical clinical demonstration of clean detachment of the periosteum from the bone with Piezotome surgery utilizing the cavitation effect: no remnants of the osteogenic layer of the periosteum are visible on the bone; the perforating blood vessels are cleanly cut.

**Figure 4:**
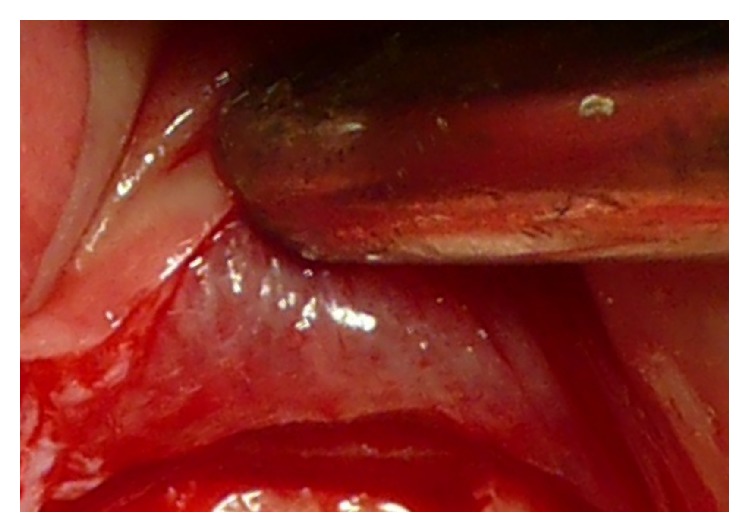
Close up view of the reverted periosteum cleanly detached from the bone with Piezotome surgery during preparation for a subperiosteal tunnel procedure. The intraperiosteal vessel-net is visible and undamaged.

To achieve a standardized reproducible clean separation of the intact periosteum from the bone (Figures 3 and 4) when creating the subperiosteal tunnel, an ultrasonic device was used (Piezotome II or Implantcenter II/Satelec-ACTEON/France) for preparation with the BS 4-tip.

**Figure 5:**
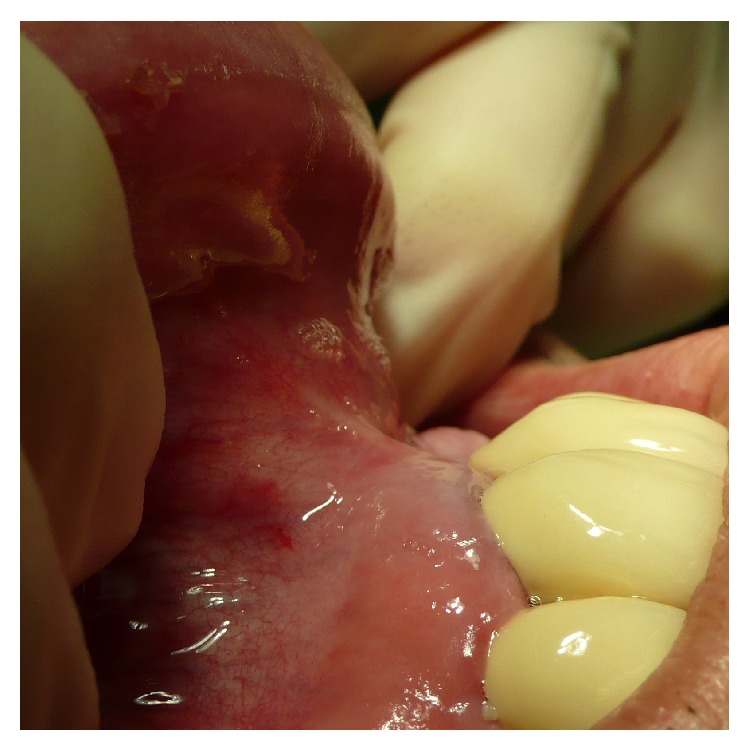
Presurgical clinical inspection of the lateral atrophic alveolar ridge.

**Figure 6:**
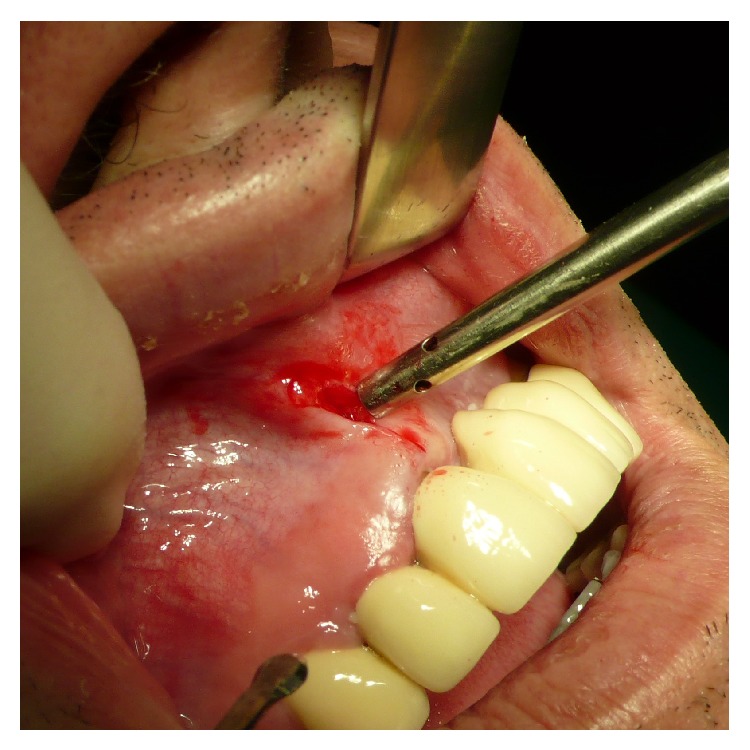
Vertical incision mesial of the augmentation site (left 1st and 2nd incisor).

**Figure 7:**
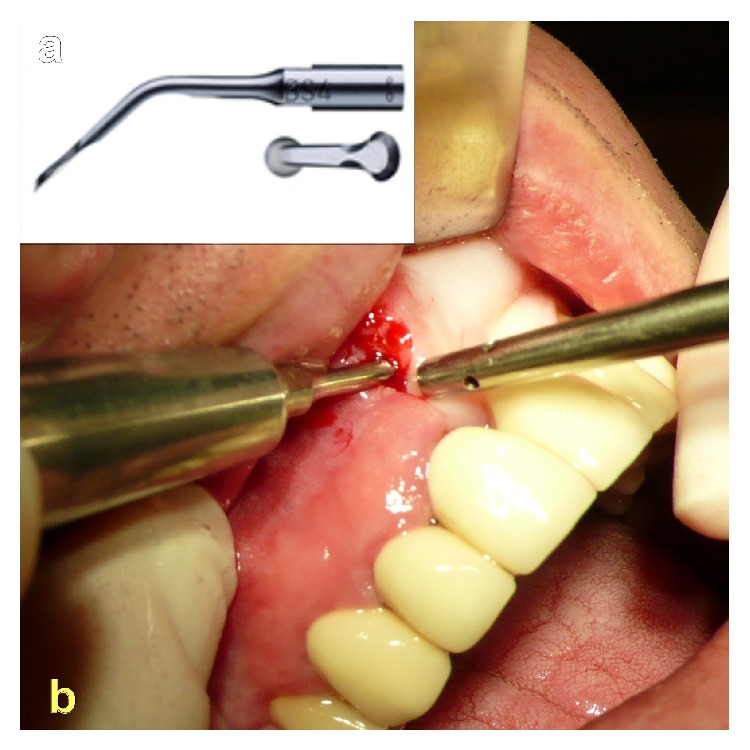
(a) BS-4 working tip for Piezotome II/SOLO/Implant Center II. (b) Clean detachment of the periosteum from the maxillary bone with the Piezotome-device up to the basal margins of the nasal cavity.

**Figure 8:**
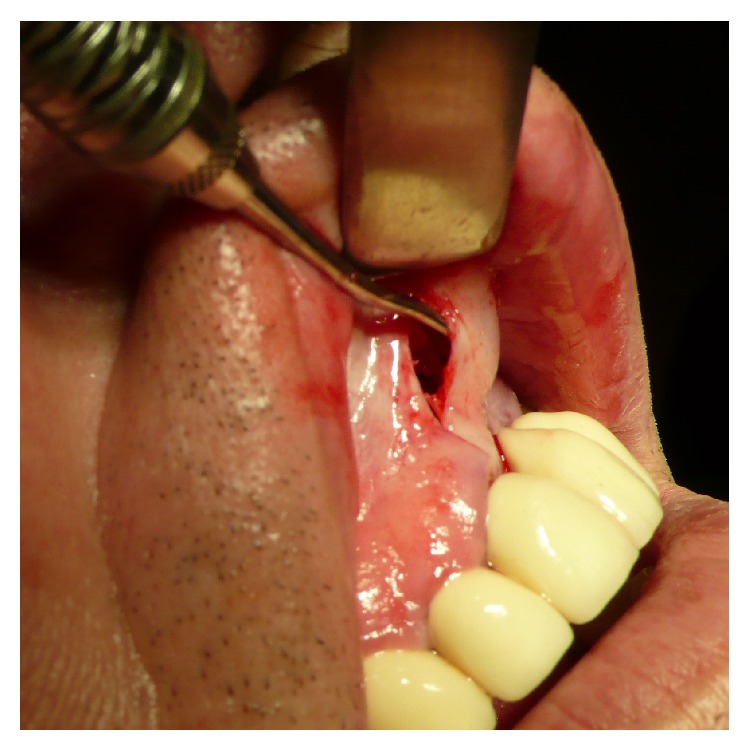
Inspection of the subperiosteal tunnel and probe-testing for sufficient pocket-extensions to the left canine-region.

**Figure 9:**
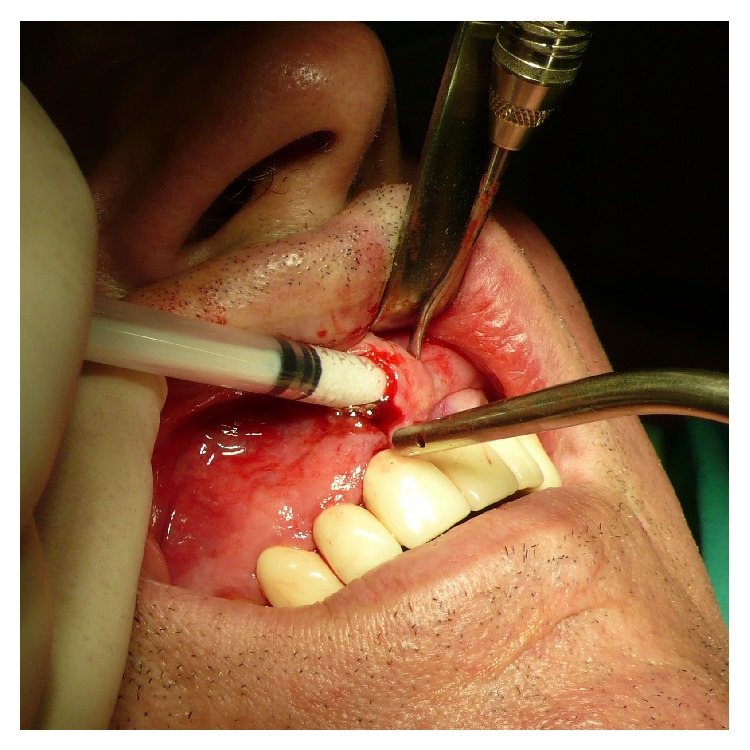
Insertion of the moldable and in situ hardening biomaterial.

**Figure 10:**
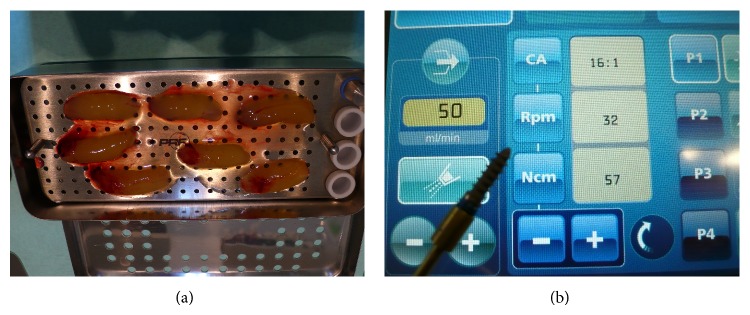
(a) Preparation of PRF to be applied to pure bTCP in situ hardening biomaterial when randomly assigned. The PRF-clots will be compressed to form an elastic membrane and the expressed liquid aspirated with a sterile syringe for later washout of the “Biolinker” to harden the biomaterial to a bone-block-like graft. By this procedure active angiogenic and osteoblast stimulating cells are perfused inside the hardened porous biomaterial block. (b) Torque measurement in 1 Ncm-steps with the Implantcenter II, implant motor.

**Figure 11:**
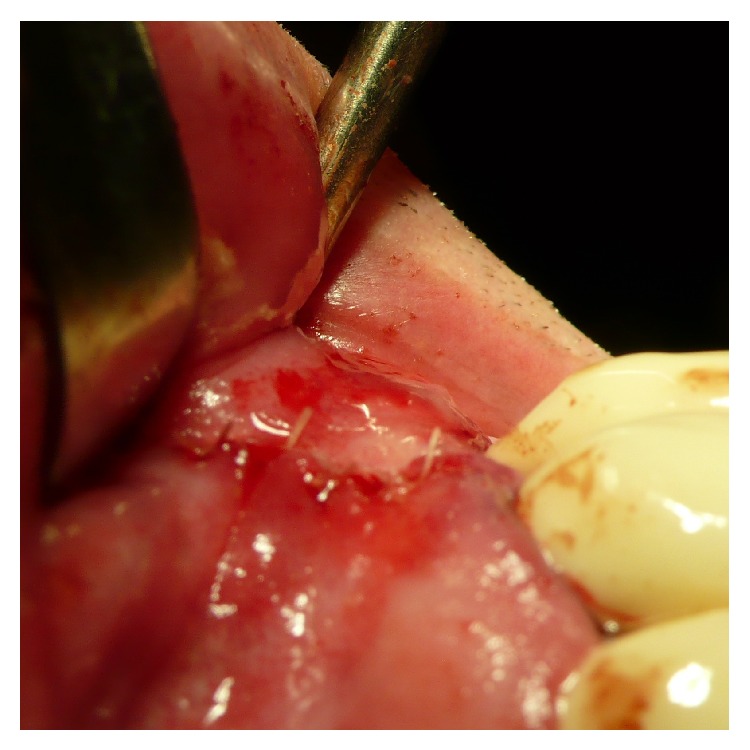
Wound closure with single stitches.

After clinical inspection (Figure 5) a vertical mucoperiosteal incision of approximately 10 mm was done 5–8 mm mesial of the augmentation site (Figure 6) followed by the preparation of the subperiosteal tunnel with the ultrasonic working-tip BS 4 (Figure 7(a)) attached to the hand piece of the Piezotome II or Implant Center II device (Figure 7(b)). The entrance of the tunnel then was checked for width (Figure 8) to allow insertion of the syringe with the biomaterial (Figure 9) and if assigned an additional layer of aPRF-membrane (Figure 10(a)). To achieve comparable and unbiased results 1 ccm of biomaterial was applied per tooth site (i.e., 1 ccm for single-tooth-gaps, 2 ccm for a gap of two missing teeth, etc.) After molding and hardening of the biomaterial by natural blood flow or injection of aPRF-liquid the vertical incision was closed by single-stitch sutures (Figure 11).

The moldable and in situ hardening biomaterial as well as the addition of aPRF was randomly assigned (Excel Random Generator formula “=RUNDEN(ZUFALLSZAHL()∗2;0)+1”) in three specifications:

1. easy-graft CRYSTAL, granule size 0.45–1 mm (SUNSTAR Degradable Solutions AG/Zurich/CH): microporous compound particles of 40% beta-tricalcium phosphate (beta-TCP) and 60% hydroxyapatite (HA), each particle covered by a 10 micrometer layer of polylactic-co-glycolic acid (PLGA); the primary loose particles are perfused with a “Biolinker” (N-methyl-2-pyrrolidone solution) and once the Biolinker is washed out by the natural blood flow the biomaterial hardens to a solid bone-substitute block (assigned implant sites: *n* = 36);
2. easy-graft CLASSIC, granule size 0.5–1 mm (SUNSTAR Degradable Solutions AG/Zurich/CH): microporous particles of pure beta-tricalcium phosphate (beta-TCP), each particle covered by a 10 micrometer layer of polylactic-co-glycolic acid (PLGA); the primary loose particles are perfused with a “Biolinker” (N-methyl-2-pyrrolidone solution) and once the Biolinker is washed out by the natural blood flow the biomaterial hardens to a solid bone-substitute block (assigned implant sites: *n* = 35);
3. easy-graft CLASSIC, granule size 0.5–1 mm (SUNSTAR Degradable Solutions AG/Zurich/CH): microporous particles of pure beta-tricalcium phosphate (beta-TCP), each particle covered by a 10 micrometer layer of polylactic-co-glycolic acid (PLGA); the primary loose particles are perfused with a “Biolinker” (N-methyl-2-pyrrolidone solution). Instead of washing out the Biolinker by natural blood flow the Biolinker was washed out forcefully with the liquid obtained in the preparation of advanced Platelet Rich Fibrin membranes (aPRF/SYFAC/France) in a sterile syringe to harden the biomaterial and a layer of aPRF was placed subperiosteal (assigned implant sites: *n* = 38).

**Figure 12:**
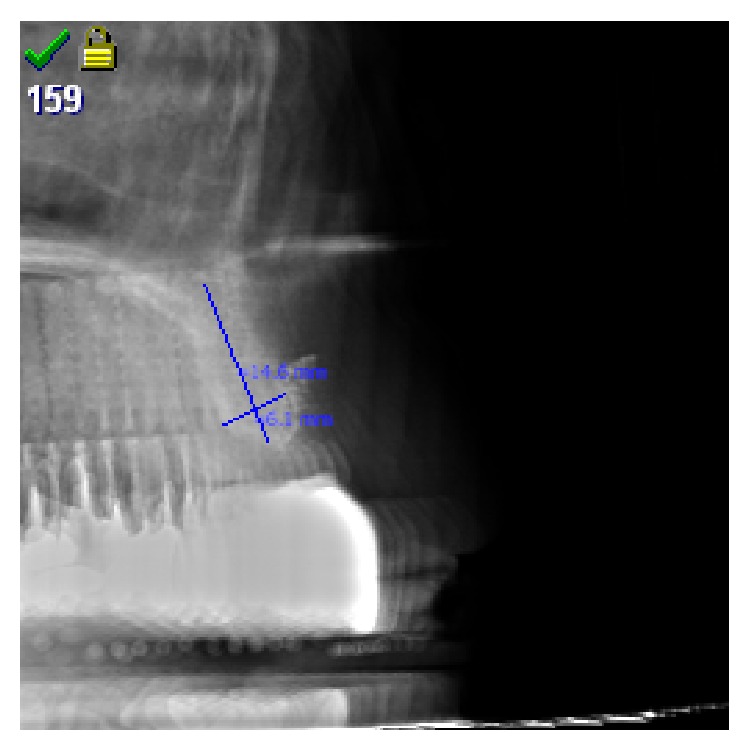
CBCT control and measurement of achieved bone dimensions prior to implant insertion.

After 6-7 months a control CBCT of the augmentation site was taken to check the bone dimensions for the planned implant insertion (Figure 12).

Following the results of a study investigating the average time for completion of bone-regeneration in sinus lift procedures [6] and to achieve an unbiased comparison with the results obtained in the sinus lift study [33] implants were inserted after an average healing time of 8.3 months (max: 8.6 months, min: 7.9 months).

**Figure 13:**
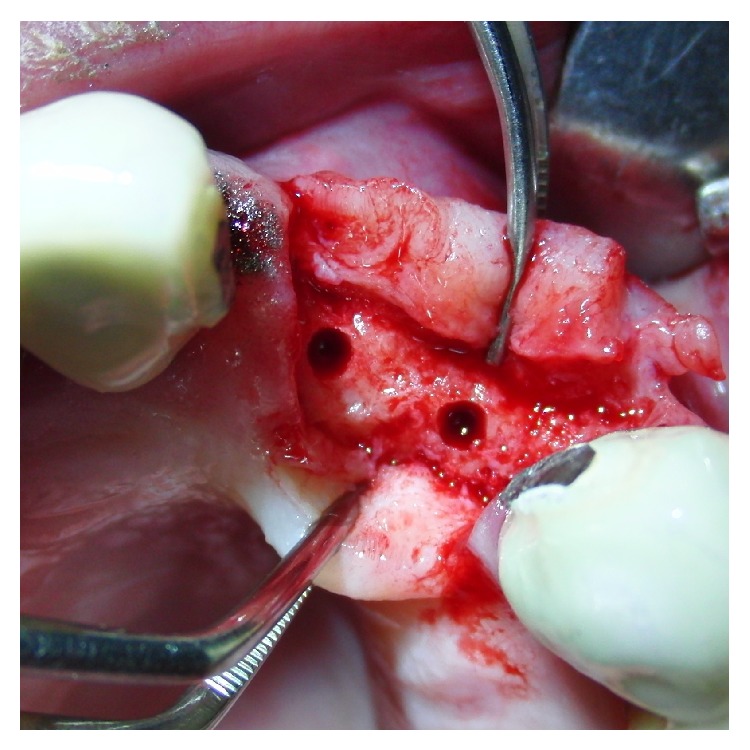
Crestal opening of the augmentation site, clinical inspection, and pilot- and form-drilling for implant insertion.

**Figure 14:**
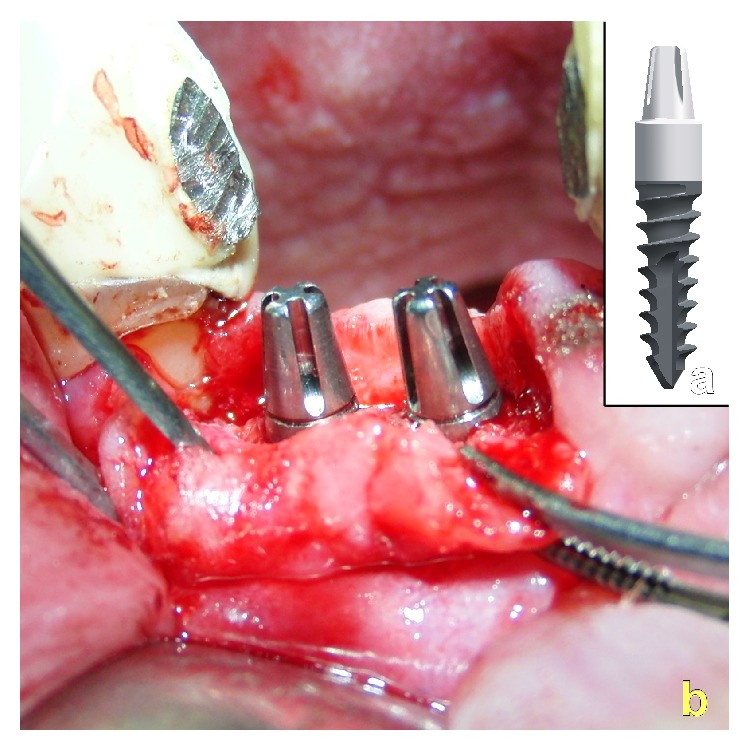
(a) Q1-Implant shape. (b) Q1-implants with a diameter of 3.5 mm and length of 14 mm inserted into the augmentation site.

In all cases one, two or three single stage Q1-implants (self-taper, root analogue screw-design; TRINON-Karlsruhe GmbH/GER) with uniform dimensions of 14 mm length and diameter 3.5 mm were inserted into the augmentation site after a top crestal incision and minimal reversion of the mucoperiosteal flaps to the buccal and palatal side for clinical inspection of the alveolar crest (Figures 13, 14(a), and 14(b)). To achieve unbiased results the Q1-implants (Figure 14(a)) were inserted by a different surgeon than the surgeon performing the subperiosteal tunnel procedure and randomly assigning the biomaterial.

All implants were inserted precisely following the drilling protocol required by the Q1-implant manufacturer with pilot and final form drilling at 50 rpm with unused drills for each implant. No specimens for histologic analysis could have been taken with a trephine-drill due to the conical shape of the final form-drill for the single-stage Q1-implant to prevent biases of ITV-measurements.

Determination of insertion-torque-value (ITV) was taken then with the Implantcenter II (Satelec-ACTEON/FR) for each inserted implant, allowing a torque increase in steps of 1 Ncm up to 100 Ncm (Figure 10(b)). Implant insertion was started with a basic torque-setting of 15 Ncm increasing in 1 Ncm-steps by an assistant until the single stage Q1-implant was inserted to its full length of 14 mm. All implants were treated with provisional, anatomical correct resin-crowns with no occlusal contacts for a period of 3 months.

Statistical evaluation was performed by a one-way ANOVA-test, Student's *t*-test, and post hoc multiple comparisons by Fisher's least significant difference (LSD) to test mean insertion-torque-values and variance in each group and mean difference significance between all groups. Additionally, the data were depicted in notched box plots to show the data distribution and interquartile ranges (IQR) between the 25th and 75th percentile of the specific biomaterial tested.

**Figure 15:**
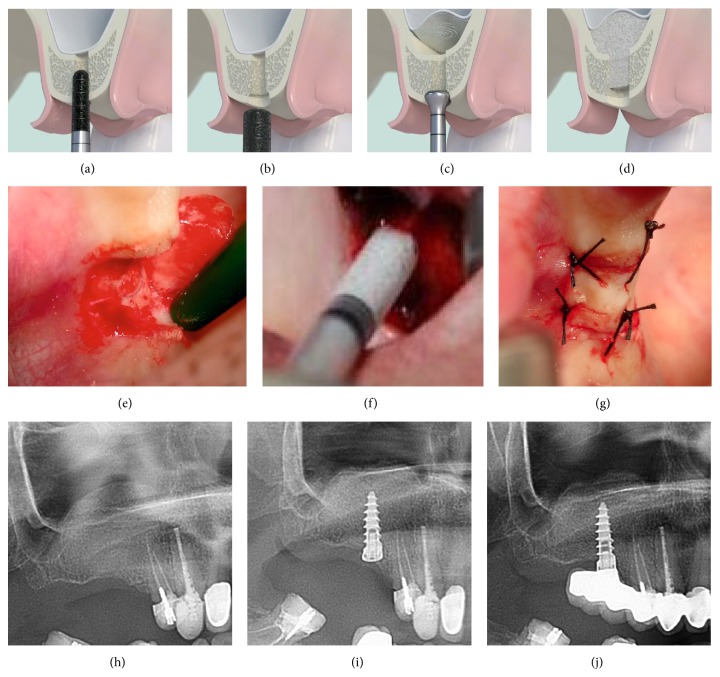
Overview tHUCSL-INTRALIFT procedure with Piezotome II/SOLO: (a) after reverting a minimal invasive crestal flap the sinus floor is opened transcrestal with the diamond coated Piezotome-tip TKW2, (b) a sealing receptacle is prepared with the diamond-coated Piezotome-tip TKW4, (c) the Piezotome-tip TKW5 is tight-fit inserted into the receptacle to prevent liquid-back-flow to the oral cavity and by this subantral loss of hydrodynamic pressure. The Piezotome then is activated and the periosteum of the Schneiderian membrane cleanly detached from the bony antrum floor utilizing the cavitation effect. (d) Insertion of 2 ccm biomaterial into the subperiosteal scaffold, (e) clinical case of a typical tHUCSL-INTRALIFT-procedure, (f) subantral application of 2 ccm biomaterial, (g) wound closure, (h) postsurgical X-ray control of the sinus lift site, (i) implant insertion after average 8.7 months, and (j) final prosthetic treatment.

The results then were compared with the results of a prior similar study investigating drill-torque and insertion-torque-values after sinus lifting with the tHUCSL-INTRALIFT-method using four different biomaterials (Bio-Oss, NanoBone, easy-graft CLASSIC, easy-graft CRYSTAL) [33]. The basic setup and healing periods for this study were the same and by this unbiased comparable to the present study. The slightly different dimension of the two-stage Q2-implant used in the prior study is compensated mathematically by a 2 mm longer length of the single-stage Q1-implant (Figure 15).

## 3. Results

All surgeries proceeded uneventful with no complications such as infections or wound dehiscences. Patients reported only minor swellings during the first 5 days postsurgical and almost no pain (average intake of Ibuprofen 400 mg mean value: 3.2 tablets, max: 6 tablets, min: 0 tablets). The patient's main complaint referred to a “voluminous feeling” beneath the lip and under the nose in the augmentation area, especially when no interim bridge was attached as preexistent or newly manufactured provisional restoration but only removable partial overdentures without any buccal resin shield.

All subperiosteal tunnel augmentation sites presented sufficient even buccal bone gain in radiographic follow-up prior to implant insertion with a minimum top-crestal ridge width of 6 mm.

**Figure 16:**
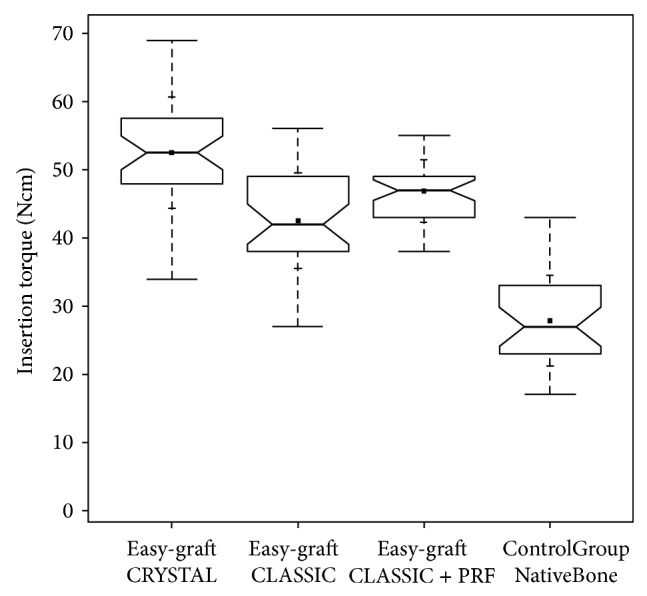
Insertion-torque-value (ITV) comparison notched box plot for the subperiosteal tunnel procedure in the anterior maxilla depicts the interquartile range (IQR) between the 25th and 75th percentile of the specific biomaterial tested where 50% of the data points were located. Additionally, the upper whiskers represent data within the 75th percentile +1.5 times the IQR. The lower whisker delimits data of the 25th percentile −1.5 times the IQR. Within the boxes the notches mark the confidence interval based on the median ± 1.58 (IQR/sqrt of *n*) (*n* = number of measurements). Additionally, the mean value is indicated by a black square and the cross symbol (+) displays the standard deviation. Easy-graft CRYSTAL (*n* = 36), easy-graft CLASSIC (*n* = 35), easy-graft CLASSIC + Platelet Rich Fibrin (*n* = 38), and control group native anterior maxillary bone (*n* = 30).

**Figure 17:**
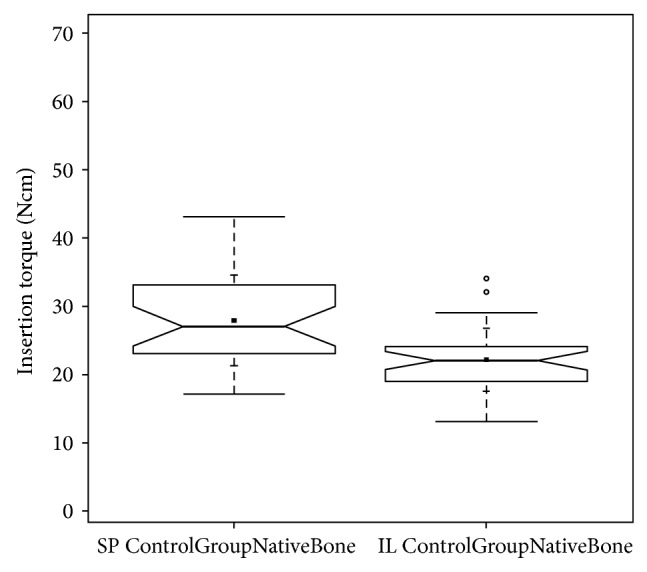
ITV-comparison notched box plot of native anterior maxillary bone (subperiosteal tunnel procedure control group—SP ControlGroupNativeBone) and native molar maxillary bone (INTRALIFT control group—IL ControlGroupNativeBone) depicts the interquartile range (IQR) between the 25th and 75th percentile of the specific biomaterial tested where 50% of the data points were located. Additionally, the upper whiskers represent data within the 75th percentile +1.5 times the IQR. The lower whisker delimits data of the 25th percentile −1.5 times the IQR. Within the boxes the notches mark the confidence interval based on the median ± 1.58 (IQR/sqrt of *n*) (*n* = number of measurements). Additionally, the mean value is indicated by a black square and the cross symbol (+) displays the standard deviation. Control group native anterior maxillary bone SP (*n* = 30), control group native molar maxillary bone IL (*n* = 35).

Insertion-torque-values (ITV) were lowest in the control group when implants were inserted into native anterior maxillary bone (mean value: 27.87 Ncm, stand. dev.: 6.66 Ncm) (Figure 16) but significantly (*P* < 0.05) higher when compared to native maxillary bone in the premolar and molar region (mean value: 22.11 Ncm, stand. dev.: 4.64 Ncm) [33] (Figure 17).

ITVs were significantly (*P* < 0.05) different between all groups and highest (mean value: 52.5 Ncm, stand. dev.: 8.15 Ncm) in the group treated with the biphasic self-hardening biomaterial (BiSHB: 60% HA, 40% bTCP) followed by the group treated with monophasic self-hardening biomaterial (MoSHB: 100% bTCP) with addition of advanced Platelet Rich Fibrin (aPRF) (mean value: 46.89 Ncm, stand. dev.: 4.57 Ncm) and without addition of aPRF (mean value: 42.51 Ncm, stand. dev.: 7.03 Ncm) and the control group of native bone (mean value: 27.87 Ncm, stand. dev.: 6.66 Ncm) (Table 1 and Figure 16).

**Figure 18:**
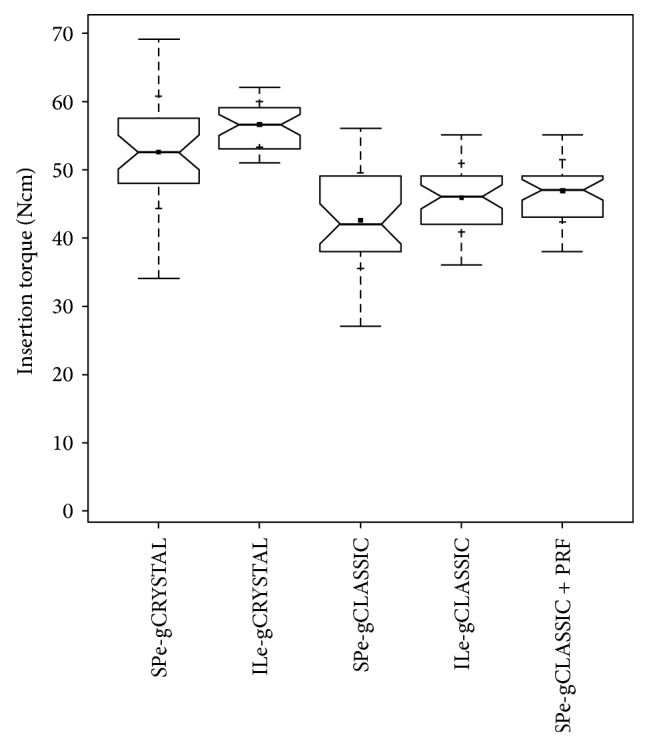
ITV-comparison notched box plot of the subperiosteal tunnel procedure (SP) in the anterior maxilla and the INTRALIFT-procedure (IL) with BiSHB (e-gCRYSTAL), MoSHB (e-gCLASSIC), and MoSHB + Platelet Rich Fibrin (PRF) (e-gCLASSIC + PRF) depicts the interquartile range (IQR) between the 25th and 75th percentile of the specific biomaterial tested where 50% of the data points were located. Additionally, the upper whiskers represent data within the 75th percentile +1.5 times the IQR. The lower whisker delimits data of the 25th percentile −1.5 times the IQR. Within the boxes the notches mark the confidence interval based on the median ± 1.58 (IQR/sqrt of *n*) (*n* = number of measurements). Additionally, the mean value is indicated by a black square and the cross symbol (+) displays the standard deviation. SPe-gCRYSTAL (*n* = 36), ILe-gCRYSTAL (*n* = 38), SPe-gCLASSIC (*n* = 35), ILe-gCLASSIC (*n* = 41), and SPe-grCLASSIC + PRF (*n* = 38).

Comparing the ITVs for BiSHB obtained with the Piezotome-enhanced subperiosteal-tunnel-technique (PeSPTT) in the anterior maxilla with the results of the tHUCSL-INTRALIFT study [33] the mean ITV values in the INTRALIFT group were significant (*P* < 0.05) higher (INTRALIFT group: mean value: 56.58 Ncm, stand. dev.: 3.36 Ncm, PeSPTT group: mean value: 52.5 Ncm, stand. dev.: 8.15 Ncm) and standard deviation significant (*P* < 0.05) lower (INTRALIFT group: stand. dev.: 3.36 Ncm versus PeSPTT group: stand. dev.: 8.15 Ncm) (Table 2 and Figure 18).

Contrary the ITVs for MoSHB with addition of aPRF were higher when used for the Piezotome-enhanced subperiosteal-tunnel-procedure in the anterior maxilla (mean value: 46.89 Ncm) compared to MoSHB without aPRF applied to INTRALIFT sites (mean value: 45.85 Ncm), but not significant (*P* = 0.41) as well as for standard deviation (PeSPTT sites: 4.57 Ncm versus INTRALIFT-sites: 5.01 Ncm) (Table 2 and Figure 18).

MoSHB without aPRF applied to PeSPTT sites revealed a significant (*P* = 0.01) lower ITV (mean value: 42.51 Ncm) compared to MoSHB applied to INTRALIFT sites (mean value: 45.85 Ncm) with a higher standard deviation (SP: 7.03 Ncm versus IL: 5.01 Ncm) (Table 2 and Figure 18).

In general, standard deviation for both BiSHB and MoSHB without aPRF in PeSPTT-sites (SP) was significantly higher than in INTRALIFT (IL) sites and subperiosteal tunnel sites (SPPRF) treated with MoSHB with aPRF (SP-BiSHB: 8.16 Ncm and SP-MoSHB: 7.03 Ncm versus IL-BiSHB: 3.6 Ncm, IL-MoSHB: 5.01 Ncm, and SPPRF-MoSHB: 4.57 Ncm) (Figure 18).

**Figure 19:**
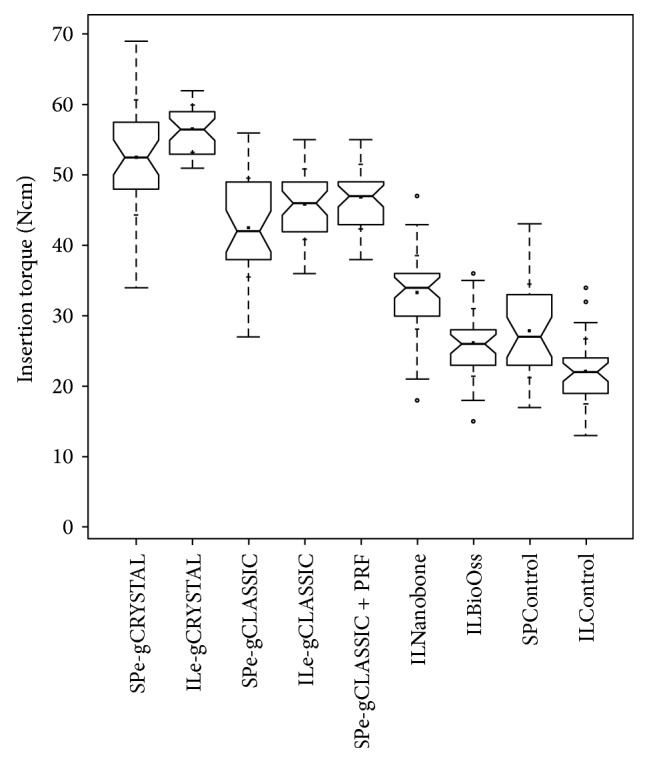
Cumulative ITV-comparison notched box plot of the subperiosteal tunnel procedure (SP) in the anterior maxilla and the INTRALIFT-procedure (IL) with BiSHB (e-gCRYSTAL), MoSHB (e-gCLASSIC), MoSHB + Platelet Rich Fibrin (PRF) (e-gCLASSIC + PRF), synthetic granulate HA/SiO_2_ (ILNanobone), bovine graft (ILBioOss), control group SP (SPControl), and control group IL (ILControl) depicts the interquartile range (IQR) between the 25th and 75th percentile of the specific biomaterial tested where 50% of the data points were located. Additionally, the upper whiskers represent data within the 75th percentile +1.5 times the IQR. The lower whisker delimits data of the 25th percentile −1.5 times the IQR. Circles (°) represent the outliers. Within the boxes the notches mark the confidence interval based on the median ± 1.58 (IQR/sqrt of *n*) (*n* = number of measurements). Additionally, the mean value is indicated by a black square and the cross symbol (+) displays the standard deviation. SPe-gCRYSTAL (*n* = 36), ILe-gCRYSTAL (*n* = 38), SPe-gCLASSIC (*n* = 35), ILe-gCLASSIC (*n* = 41), SPe-grCLASSIC + PRF (*n* = 38), ILNanobone (*n* = 42), ILBioOss (*n* = 34), SPControl (*n* = 30), and ILControl (*n* = 35).

An overall cumulative comparison between the tested loose granule biomaterials (bovine bone: BioOss, synthetic graft: Nanobone 100% HA embedded in a SiO_2_-matrix) for the INTRALIFT procedure and MoSHB and BiSHB applied in both INTRALIFT and PeSPTT procedures (Table 2) is depicted in Figure 19. No statistical significant differences were found when the ITVs of the control group in the anterior maxilla are compared with INTRALIFT sites augmented with bovine bone (*P* = 0.2) and MoSHB + aPRF in PeSPTT sites with INTRALIFT sites augmented with MoSHB without aPRF (*P* = 0.4).

## 4. Discussion

The physiological cycle of bone remodeling is a continuous event and is adjusted by peri- and endosteum [44]. Bone mass and mechanical strength increases on continuous demand to bear higher loads or decreases down to atrophy by lack of physiological load as given in an edentulous alveolar crest or general or bone-specific diseases [32]. Muscles, tendons, teeth, periosteum, and endosteum are intimately connected to and anchored in the calcified structure of bone by elastin-rich collagenous Sharpey fibres which “integrate into a periosteal Sharpey fiber-endosteum (PSE) structural continuum” allowing muscular forces to act on bone in its function as lever for movement withstanding static and dynamic loading forces. The same principle is valid for the elastin-rich Sharpey fibres of the periodontal ligament that interweaves the entire alveolar ridge [45]. The maintenance of the alveolar ridges is the result of functional loaded teeth within its “biological load width” and not its precondition.

The elastin-rich Sharpey fibres too are most suitable to distribute and dampen the strain on the calcified structures of bone. This “periosteal Sharpey fiber-endosteum (PSE) structural continuum” together with the connected “acting” organs (muscles, tendons, and teeth) shows an efficient organizational pattern relative to forces introduced. By this, Sharpey fibres are suggested as main peri- and endosteal trigger for increase of bone mass and biomechanical stability on continuous higher loads by stimulation of osteoblasts originating from the cambium layer of peri- and endosteum [45]. Contrary, a lack of forces introduced into bone by the PSE-structural continuum (as a reaction to physical inactivity or in extreme, e.g., during spaceflights by the lack of gravity) leads to disorganization of the Sharpey fibres followed by a decrease of calcified bone mass down to atrophy [46, 47] proving the vital role of peri- and endosteum in bone maintenance, repair, and regeneration. Chronic overload exceeding the “biological biomechanical resistance width” leads to bone resorption and possible loss of osseointegrated (dental) implants too but is used therapeutically in orthodontics. Compared to long bones the mandible contains a greater amount of collagen and by its structural specifics “likely renders more flexibility to the bone and leaves it more suited to constant exercise,” remodeling and higher load bearing [48] which might probably be supportive for GBR procedures in CMF and oral surgery.

But also among the jawbones, the maxilla and the mandible, significant differences can be found: the mandibular alveolar crest provides a 2.8-fold greater bone volume compared to the maxillary alveolar crest and shows a significant and, regarding anterior and posterior sections, uniformly higher bone formation rate compared to the maxilla, whereas in the maxillary alveolar crest significant differences of bone formation can be found in the anterior section compared to the posterior maxilla [18]. These experimental results obtained in an animal experiment can be backed now clinically in humans when the ITVs of natural subantral bone are compared with ITVs in the anterior maxilla (Figure 17).

While the PSE structural continuum mainly maintains crestal bone and induces bone regeneration, surgical GBR techniques, mostly creating “critical size defects” far above the biological threshold [33], demand a proper immobilization beyond the biological threshold [29] of the augmented scaffold* in toto* to allow the first step in bone restoration: proper vascularization [35] and osseointegration of biomaterials [30] and/or implants. This mandatory immobilization of the augmentation scaffold to prevent fibrous tissue encapsulation of the implanted grafts [27–29] can be obtained by traditional surgical methods: free bone block grafts, harvested from the chin or lateral ramus of the mandible, are rigidly fixed to the augmentation site with osteosynthesis screws. These procedures need the preparation of vast full thickness mucoperiosteal flaps and by this the partial destruction of the periosteum and its vascularization by periosteal slotting to achieve a tensionless wound closure. Vascularization of the augmentation site is massively obstructed by the surgical procedure itself. A high risk of multiple complications such as voluminous postsurgical edema (which by itself diminishes blood circulation in the surgical site), total, or partial resorption of the graft by lack of vascularization, wound dehiscence, and donor-site complications are commonly known to accompany these procedures.

Contrary, the results of the present and similar prior studies [31, 33, 37–39] suggest minimal invasive surgical procedures such as the tHUCSL-INTRALIFT [7] or the subperiosteal-tunnel-technique (SPTT) to preserve the functional integrity of the periosteum [6, 7, 33] (Figures 1 and 3).

Obviously the use of xenogeneic bovine, synthetic HA and the new class of moldable, and self-hardening bone grafts (monophasic bTCP {MoSHB} or biphasic HA/bTCP {BiSHB}) in guided-bone-regeneration procedures significantly enhances the (bio)mechanical stability of the restored anterior and subantral maxillary alveolar crest (Figure 19), but by a twofold when BiSHB is used.

Contrary to native subantral maxillary bone compared to the anterior alveolar crest, the resulting (bio)mechanical stability after augmentation with BiSHB (60% HA/40% bTCP) and MoSHB (100% bTCP), expressed by higher ITVs, reveals to be significantly higher with a significant lesser variance in tHUCSL-INTRALIFT sites compared to the subperiosteal-tunnel-augmentations in the anterior maxilla (Figure 18). Having the same maxillary bone-base and a comparable geometrical subperiosteal scaffold containing comparable volumes of BiSHB or MoSHB (Figure 2) the ITVs in the anterior maxilla should be at least equal to the tHUCSL-INTRALIFT sites if not higher because of the natural higher bone reformation in the anterior maxilla [18].

A hypothesis to explain these differences could be the substantially different forces acting on the augmentation sites in the timeline of the bone regeneration cycle. While in sinus-lifting sites the only recurrent force is the breathing activity creating only linear vertical pressure differences of average 15 mbar with multiple single or multiple peak-pressure changes of 1.5 bar in sneezing activities [33] the subperiosteal augmentation site in the anterior maxilla is exposed to possibly much higher and omnidirectional forces by speaking, smiling, and food-intake activities. This possibly leads to micromotions in the periosteum BiSHB and MoSHB graft interface and in the BiSHB and MoSHB bone interface that exceed the biological threshold [27–29] and lead to a reduced angiogenesis [35]. This plausible assumption could lead to the observed lesser (bio)mechanical stability of the augmented maxillary bone in the anterior maxilla and might also explain why clinical trials with loose granule biomaterials (xenogeneic and synthetic of various brands) inserted into subperiosteal scaffolds in the anterior maxilla following the procedures described in the literature only for the mandible [19–24] failed from the beginning, led to mere fibrous encapsulation as with nonprimary stable loaded dental implants [29], and had to be canceled. On the other hand, once advanced Platelet Rich Fibrin (aPRF) is added to the MoSHB graft the (bio)mechanical stability of the anterior PeSPTT site slightly supersedes the ITVs obtained in the sinus lift study (Figure 18) and by this strongly backs the evidence, in which PRF induces a faster and better angiogenesis and bone reformation [36–39] alleviating the effects of PeSPTT surgical site micromotions above the biological threshold.

As experimental [30, 31], clinical [33], and the presented studies furthermore suggest, biomaterials containing hydroxyapatite (HA) seem to allow a more mechanical stable bone restoration superior to fast resorbing pure bTCP and to native jawbone, which in the clinical routine is highly welcome for later implant insertion (Figure 19). This fact might be attributed to the very slow resorbing structural dense biochemical nature of HA acting as structural enforcement of the surrounding restored native bone once fully osseointegrated comparable to the tubular HA architecture of the mammal long bones (the tubular HA architecture of long bones (osteon) represents a higher evolutionary stage than the woven bones of the skull and ribs). For the clinician an additive use of BiSHB grafts in conjunction with aPRF for both PeSPTT and tHUCSL-INTRALIFT seems to be preferable in order to achieve the highest possible biomechanical stability of the restored alveolar crest and is currently under clinical investigation by the authors as an extension of the presented study.

## 5. Conclusions

Following the present knowledge of the structure, biomechanics, and physiology of bone regeneration and interpreting the results of the current and prior clinical studies [33], reproducible high clinical success rates in guided bone regeneration and implantology with least patient morbidity depend on several decisive factors in the clinical routine with distinct clinical recommendations and conclusions in respect to the knowledge of the biology of bone healing, regeneration, and restoration.

### 5.1. Atraumatic and Precise Surgery on Macroscopic and Microscopic Level

Since interruption of vascularization might lead to insufficient angiogenesis in the mostly vast augmentation sites counting up to necessary bone volumes of 2 ccm which are beyond “critical size defects” [34], as least blood vessels as possible should be interrupted by avoidance of full-thickness mucoperiosteal flaps in favor of subperiosteal tunnel or pocket techniques.

The peri- and endosteum-connecting Sharpey fibers should be cleanly cut instead of ripped and torn preferably with the aid of ultrasonic surgical tools (“Piezotomes”) that utilize the cavitation effect for a clean periosteal detachment macroscopically (Figures 3 and 4) and microscopically (Figure 1) [7], leaving the cambium layer (“cradle of osteoblasts”) undisrupted and fully intact on the cellular and functional level. Piezotome surgery is now widely suggested to generally preserve soft tissues and avoid mechanical damage [49–51], reduce tissue ischemia [52], and stimulate mesenchymal stem cell differentiation and bone healing [53–55].

The consistent results of the present study in company with the results of prior similar studies [17, 33, 50, 51] seem to support the suggestion that utilizing Piezotome surgery for preparation of the augmentation site might be the first step to achieve constant high success rates and does not require extensive manual training of the surgeon compared to traditional complex and manually challenging surgical techniques.

### 5.2. Sufficient Immobilization of the Augmentation Site

Surgical created subperiosteal and endosteal scaffolds for bone augmentation have to be perceived by the clinician more as “fracture sites” similar to “natural” fractures of the jawbones and its mandatory immobilization for proper bone healing. Minimal invasive surgical techniques such as tHUCSL-INTRALIFT [56] and the Piezotome-enhanced subperiosteal tunnel or pocket technique (PeSPTT) do not allow the use of autografted, xenogeneic, or synthetic solid bone blocks since they have to be stabilized by osteosynthesis screws. Loose autologous, xenogeneic, or synthetic granulate biomaterials are prone to macro- and micromovements by muscular, breathing (in case of the maxillary sinus), and/or soft tissue forces in the normal function of the stomatognathic and respiratory system and by this might counteract the proper immobilization of the augmentation scaffold as it was suggested by the results presented in a prior study [33]. Therefore the new class of in situ moldable and self-hardening biomaterials used in this study seems to be highly suitable in minimal invasive guided bone regeneration to achieve reproducible good results providing a sufficient and (bio)mechanical highly stable dental implant site of sufficient dimensions. This class of biomaterials seems to fulfill the biological requirement of proper immobilization of the augmentation scaffold for an undisturbed natural bone regeneration below the proposed threshold of micromobility of 100–150 *μ*m [29] in its own final bone-block-like microporous consistence and its immobility on the bone and periosteal surface.

### 5.3. Use of Biologic Active Autologous Agents Such as Platelet Rich Plasma (PRP) or Advanced Platelet Rich Fibrin (aPRF)

It can be considered as a biological fact that all biocompatible materials (i.e., titanium, ZiO, PEEK, lactic-acid polymers, bioglasses, bTCP, and natural or synthetic hydroxyapatite, etc.) are allowed to osseointegrate but are not “osteoinductive/conductive”* per se*. Differences between various origins (human, bovine, equine, porcine, all deriving from the same evolutionary stem of mammals, algae, ceramics, glasses, bTCP, and HA) and brands of biomaterials in experimental microscopic, radiologic, clinical, and statistical evaluation on resulting bone regeneration outcome rarely have a significant impact on the work of the clinician since both animal and clinical human studies almost never report if the basal cambium layer of the periosteum was intact and if and how the periosteum was slotted to achieve a tensionless wound closure.

While the postulated coverage of any augmentation site with barrier membranes of different chemical origin (PTFE, titanium, polylactic acid, collagen, etc.) is still favorable in cases of preexisting and/or iatrogenic devastation and destruction of the periosteum, it should be considered as contraindication when the periosteum is detached without lesions from the bone [57].

The better alternative in both cases could be the general replacement of barrier membranes by autologous aPRF membranes that provide a proven physiological enhancement of angiogenesis and bone growth [36–39], copying, and, by concentrating the active biological agents through centrifugation, multiplying the effect of the natural fibrin-condensation on and in every soft- and hard-tissue wound and biocompatible implant materials. Contrary to the production of PRP-concentrates the preparation of aPRF integrates easily in the timely flow of GBR surgeries and does not constitute a challenge to the performing surgeon.

The results of the current study back the experimental findings concerning the advantages for more reliable bone regeneration when aPRF is used and suggest aPRF to enhance biomechanical bone quality to a constant higher level in the clinical routine in conjunction with Piezotome surgery and BiSHB grafts to possibly achieve better and more consistent results with less patient's morbidity compared to traditional methods.

### 5.4. Paradigm Change of Understanding Guided Bone Regeneration Procedures in the Clinical Practice

Oral surgeons are suggested to turn away from a (bio)material and technical surgical instruments centered view back to a more general view on GBR surgeries based on the biological and physiological facts of bone-healing mechanisms. As it is in diagnostics and surgical planning, plain clinicians rely more and more on prefabricated recipes and software-based surgical “aids” losing out of sight the individual stomatognathic dynamics of the individual patient. Surgical planning and surgery performance should strictly follow the guidelines given by biology and dynamics of the stomatognathic system instead of prefabricated “surgical templates.” The suggestion to define autologous bone grafts as “gold-standard” in dental regenerative therapies cannot be backed any more [58] and it should stay a philosophical discussion whether regenerated/augmented bone should be only native bone or an obviously mechanical more resistive compound of native bone and synthetic biomaterials (“foreign body argument”) to sustain the introduced forces by other “foreign bodies,” that is, dental implants (made of titanium, ZiO, PEEK, etc.).

## Figures and Tables

**(a):** 

Means comparisons	

Comparisons for each pair using Student's *t*	

Confidence quantile	
*t*	Alpha

1.97769	0.05

**(b):** 

LSD threshold matrix				
Abs(Dif)-LSD				

	Easy-graft CRYSTAL_SP	Easy-graft CLASSIC + PRF_SP	Easy-graft CLASSIC_SP	Control Group Native Bone Front-Canine_SP

Easy-graft CRYSTAL_SP	−3.12	2.52	6.84	21.36
Easy-graft CLASSIC + PRF_SP	2.52	−3.04	1.28	15.79
Easy-graft CLASSIC_SP	6.84	1.28	−3.17	11.35
ContrGroupNativeBone Front-Canine_SP	21.36	15.79	11.35	−3.42

Positive values show pairs of means that are significantly different.

**(c):** 

Connecting letters report					
level	Mean

Easy-graft CRYSTAL_SP	A				52.50
Easy-graft CLASSIC + PRF_SP		B			46.89
Easy-graft CLASSIC_SP			C		42.51
ContrGroupNativeBone Front-Canine_SP				D	27.87

Levels not connected by same letter are significantly different.

**(d):** 

Ordered differences report							
Level	compared to	Level	Difference	Std. err. dif.	Lower CL	Upper CL	*P* value

Easy-graft CRYSTAL_SP		ContrGroupNativeBone Front-Canine_SP	24.63	1.66	21.36	27.91	<0.0001
Easy-graft CLASSIC + PRF_SP		ContrGroupNativeBone Front-Canine_SP	19.03	1.64	15.79	22.27	<0.0001
Easy-graft CLASSIC_SP		ContrGroupNativeBone Front-Canine_SP	14.65	1.67	11.35	17.95	<0.0001
Easy-graft CRYSTAL_SP		easy-graft CLASSIC_SP	9.99	1.59	6.84	13.13	<0.0001
Easy-graft CRYSTAL_SP		easy-graft CLASSIC + PRF_SP	5.61	1.56	2.52	8.69	0.0005
Easy-graft CLASSIC + PRF_SP		easy-graft CLASSIC_SP	4.38	1.57	1.27	7.49	0.0060

**(a):** 

Means comparisons	

Comparisons for each pair using Student's *t*	

Confidence quantile	
*t*	Alpha

1.9674	0.05

**(b):** 

LSD threshold matrix									
Abs(Dif)-LSD									

	Easy-graft CRYSTAL_IL	Easy-graft CRYSTAL_SP	Easy-graft CLASSIC + PRF_SP	Easy-graft CLASSIC_IL	Easy-graft CLASSIC_SP	Nanobone_IL	Control Group Native Bone Front-Canine_SP	BioOss_IL	Control Group Native Bone premolar-molar_IL

Easy-graft CRYSTAL_IL	−2.53	1.51	7.15	8.24	11.48	20.80	26.02	27.77	31.88
Easy-graft CRYSTAL_SP	1.51	−2.60	3.04	4.12	7.36	16.68	21.90	23.65	27.76
Easy-graft CLASSIC + PRF_SP	7.15	3.04	−2.53	−1.45	1.79	11.11	16.33	18.08	22.19
Easy-graft CLASSIC_IL	8.24	4.12	−1.45	−2.44	0.80	10.12	15.33	17.09	21.20
Easy-graft CLASSIC_SP	11.48	7.36	1.79	0.80	−2.64	6.68	11.90	13.65	17.76
Nanobone_IL	20.80	16.68	11.11	10.12	6.68	−2.41	2.80	4.56	8.67
ContrGroupNativeBone Front-Canine_SP	26.02	21.90	16.33	15.33	11.90	2.80	−2.85	−1.11	3.00
BioOss_IL	27.77	23.65	18.08	17.09	13.65	4.56	−1.11	−2.68	1.43
ContrGroupNativeBone premol-molar_IL	31.88	27.76	22.19	21.20	17.76	8.67	3.00	1.43	−2.64

Positive values show pairs of means that are significantly different.

**(c):** 

Connecting letters report								
Level								Mean

Easy-graft CRYSTAL_IL	A							56.58
Easy-graft CRYSTAL_SP		B						52.50
Easy-graft CLASSIC + PRF_SP			C					46.89
Easy-graft CLASSIC_IL			C					45.85
Easy-graft CLASSIC_SP				D				42.51
Nanobone_IL					E			33.31
ContrGroupNativeBone Front-Canine_SP						F		27.87
BioOss_IL						F		26.21
ContrGroupNativeBone premol-molar_IL							G	22.11

Levels not connected by same letter are significantly different.

**(d):** 

Ordered differences report							
Level	compared to	Level	Difference	Std. err. dif.	Lower CL	Upper CL	*P*-value

Easy-graft CRYSTAL_IL		ContrGroupNativeBone premolar-molar_IL	34.46	1.32	31.88	37.05	<0.0001
Easy-graft CRYSTAL_SP		ContrGroupNativeBone premolar-molar_IL	30.39	1.33	27.76	33.01	<0.0001
Easy-graft CRYSTAL_IL		BioOss_IL	30.37	1.33	27.77	32.98	<0.0001
Easy-graft CRYSTAL_IL		ContrGroupNativeBone Front-Canine_SP	28.71	1.37	26.01	31.41	<0.0001
Easy-graft CRYSTAL_SP		BioOss_IL	26.29	1.34	23.65	28.94	<0.0001
Easy-graft CLASSIC + PRF_SP		ContrGroupNativeBone premolar-molar_IL	24.78	1.32	22.19	27.37	<0.0001
Easy-graft CRYSTAL_SP		ContrGroupNativeBone Front-Canine_SP	24.63	1.39	21.90	27.36	<0.0001
Easy-graft CLASSIC_IL		ContrGroupNativeBone premolar-molar_IL	23.74	1.29	21.20	26.28	<0.0001
Easy-graft CRYSTAL_IL		Nanobone_IL	23.27	1.26	20.80	25.74	<0.0001
Easy-graft CLASSIC + PRF_SP		BioOss_IL	20.69	1.33	18.08	23.30	<0.0001
Easy-graft CLASSIC_SP		ContrGroupNativeBone premolar-molar_IL	20.40	1.34	17.76	23.04	<0.0001
Easy-graft CLASSIC_IL		BioOss_IL	19.65	1.30	17.09	22.21	<0.0001
Easy-graft CRYSTAL_SP		Nanobone_IL	19.19	1.28	16.68	21.70	<0.0001
Easy-graft CLASSIC + PRF_SP		ContrGroupNativeBone Front-Canine_SP	19.03	1.37	16.33	21.73	<0.0001
Easy-graft CLASSIC_IL		ContrGroupNativeBone Front-Canine_SP	17.99	1.35	15.33	20.64	<0.0001
Easy-graft CLASSIC_SP		BioOss_IL	16.31	1.35	13.65	18.97	<0.0001
Easy-graft CLASSIC_SP		ContrGroupNativeBone Front-Canine_SP	14.65	1.40	11.90	17.40	<0.0001
Easy-graft CRYSTAL_IL		Easy-graft_CLASSIC_SP	14.06	1.32	11.48	16.65	<0.0001
Easy-graft CLASSIC + PRF_SP		Nanobone_IL	13.59	1.26	11.11	16.06	<0.0001
Easy-graft CLASSIC_IL		Nanobone_IL	12.54	1.23	10.12	14.97	<0.0001
Nanobone_IL		ContrGroupNativeBone premolar-molar_IL	11.20	1.28	8.67	13.72	<0.0001
Easy-graft CRYSTAL_IL		Easy-graft CLASSIC_IL	10.73	1.26	8.24	13.21	<0.0001
Easy-graft CRYSTAL_SP		Easy-graft CLASSIC_SP	9.99	1.33	7.36	12.61	<0.0001
Easy-graft CRYSTAL_IL		Easy-graft CLASSIC + PRF_SP	9.68	1.29	7.15	12.22	<0.0001
Easy-graft CLASSIC_SP		Nanobone_IL	9.20	1.28	6.68	11.73	<0.0001
Nanobone_IL		BioOss_IL	7.10	1.30	4.56	9.65	<0.0001
Easy-graft CRYSTAL_SP		Easy-graft CLASSIC_IL	6.65	1.28	4.12	9.17	<0.0001
ContrGroupNativeBone Front-Canine_SP		ContrGroupNativeBone premolar-molar_IL	5.75	1.40	3.00	8.50	<0.0001
Easy-graft CRYSTAL_SP		Easy-graft CLASSIC + PRF_SP	5.61	1.31	3.04	8.17	<0.0001
Nanobone_IL		ContrGroupNativeBone Front-Canine_SP	5.44	1.34	2.80	8.08	<0.0001
Easy-graft CLASSIC + PRF_SP		Easy-graft CLASSIC_SP	4.38	1.32	1.79	6.97	0.0010
BioOss_IL		ContrGroupNativeBone premolar-molar_IL	4.09	1.35	1.43	6.75	0.0027
Easy-graft CRYSTAL_IL		Easy-graft CRYSTAL_SP	4.08	1.31	1.51	6.65	0.0019
Easy-graft CLASSIC_IL		Easy-graft CLASSIC_SP	3.34	1.29	0.80	5.88	0.0102
ContrGroupNativeBone Front-Canine_SP		BioOss_IL	1.66	1.41	−1.11	4.43	0.2385
Easy-graft CLASSIC + PRF_SP		Easy-graft CLASSIC_IL	1.04	1.26	−1.45	3.53	0.4108
